# Effect of Layer-Wise Varying Parameters on the Microstructure and Soundness of Selective Laser Melted INCONEL 718 Alloy

**DOI:** 10.3390/ma12132165

**Published:** 2019-07-05

**Authors:** Xiang Wang, Jinwu Kang, Tianjiao Wang, Pengyue Wu, Tao Feng, Lele Zheng

**Affiliations:** 1 School of Materials Science and Engineering, Key Laboratory for Advanced Materials Processing Technology, Tsinghua University, Beijing 100084, China; 2 Department of Mechanical and Energy Engineering, Southern University of Science and Technology, Shenzhen 518055, China; 3 Beijing e-Plus 3D Tech. Co., Ltd., Beijing 100027, China; 4 School of Mechanical, Electronic and Control Engineering, Beijing Jiaotong University, Beijing 100044, China

**Keywords:** selective laser melting, microstructure, defects, Inconel 718, laser energy density

## Abstract

Selective laser melting (SLM) is a promising powder bed fusion additive manufacturing technique for metal part fabrication. In this paper, varying scanning speed in the range of 500 mm/s to 1900 mm/s, and laser power in the range of 100 W to 200 W, were realized from layer to layer in a cycle of 56 layers in a single cuboid Inconel 718 alloy specimen through SLM. Layer-wise variation of microstructure and porosity were acquired, showing the layer-wise controlling capability of microstructural soundness. The melt pool size and soundness are closely linked with the energy input. High energy density led to sound regions with larger, orderly stacked melt pools and columnar grains, while low energy density resulted in porous regions with smaller, mismatched melt pools, un-melted powder, and equiaxed grains with finer dendrites. With the increase of laser energy density, the specimen shifts from porous region to sound region within several layers.

## 1. Introduction

Selective laser melting (SLM) is a type of powder bed fusion additive manufacturing technology, which melts metal powder using tiny a laser spot track-by-track, layer-by-layer to form metal components [1,2]. Due to this unique feature of SLM, it is capable of controlling the microstructure of any location of a part. Laser power (*P*), scanning speed (*v*), hatch spacing (*d*), and layer thickness (*t*) are the main process parameters. Laser energy density, *E* = *P*/(*v*·*d*·*t*), is used to regulate the microstructure and mechanical properties [3,4]. Studies conducted in the past have shown that, with the increase in energy density, soundness and mechanical properties are significantly enhanced [5,6,7]. Swee et al. [8] conducted experiments on the selective laser melting of TiTa alloy and concluded that increasing laser energy density is needed to achieve fully dense parts and to fully melt the tantalum in the powder. Additionally, Liu et al. [9] revealed that high energy input also leads to higher proportion of columnar grains. Yu et al. [10] applied re-melting on the SLM of AlSi10Mg and found that it reduced surface roughness as well as the porosities in the as-printed specimens. Grain morphologies and texture can also be altered by different scan strategies. Wan et al. [11] found that a 90° rotation of scan directions leads to stronger cubic texture because of the direction change of heat flux. Kurzynowski et al. [12] discovered that perpendicularly re-melting the current layer alters the microstructural texture from cubic to partially fiber. Kirka et al. [13] adopted a point heat source strategy in electron beam melting (EBM) and realized either columnar or equiaxed grain structures upon solidification through changes in the process parameters for nickel-base super-alloy. Studies with post-process treatments were also performed on selective laser melted samples. Mostafa et al. [14] studied the structure and texture of selective laser melted Inconel 718 alloy and concluded that the columnar grains, with strong {100} texture in the as-printed sample, shifted to equiaxed grains after heat treatment. Calandri et al. [15] adopted heat treatment on selective laser melted Inconel 718 alloy in different temperatures and reached maximal hardness value by aging at 714 °C.

However, most studies are limited to one specimen out of one set of parameters. In this paper, specimens with a wide range of varying parameters were fabricated by selective laser melting to gain a thorough understanding of the effect of these parameters on defects and microstructure. This has the potential of bringing out the uniqueness of SLM to achieve a microstructural gradient in one component.

## 2. Materials and Methods

**Figure 1:**
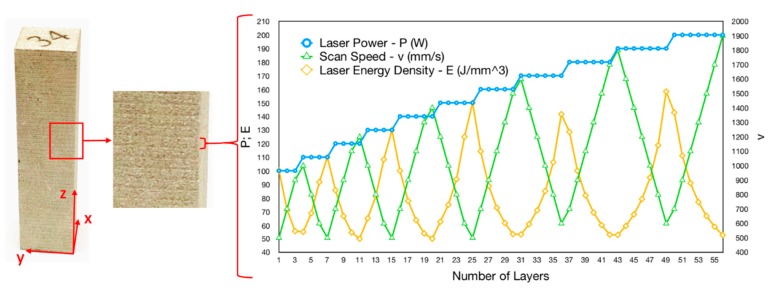
As-printed specimen and the applied parameters of each layer in a cycle.

Nickel-based Inconel 718 alloy was selected for the experiment. Due to its excellent mechanical properties under a wide range of temperature, it has long been considered as one of the most highly applicable materials for gas turbine blades, aero-engines and other parts in the field of aeronautics. Pre-alloyed gas-atomized Inconel 718 powder, produced by AMC Powders Co., Ltd. (Beijing, China), was used. Cuboid specimens of 15 × 15 × 60 mm^3^ were fabricated using the SLM based 3D printer EP-M100T manufactured by Beijing e-Plus 3D Tech. Co., Ltd. (Beijing, China). An Al substrate was applied. Highly pure argon gas was introduced to lower oxygen contamination. Raster scan strategy with a rotation of 90° in consecutive layers was adopted. The as-printed specimen with the applied parameters is shown in Figure 1. With the layer thickness (*t*) and hatch distance (*d*) held constant at 20 µm, and 100 µm, respectively, laser power (*P*) and scanning speed (*v*) varied from layer to layer repeatedly in every 56 layers. The laser power applied increased stepwise from 100 W to 200 W for every 3–6 layers, while scan speed changed back and forth from 500 mm/s to 1900 mm/s. Thus, the volume energy density shifted from 50–150 J/mm^3^.

Specimens were cut and then ground with 240–2000 waterproof abrasive papers and polished with diamond suspension from 2 mm to 0.5 mm and with colloidal silica suspension of 0.05 mm, then electrolytically etched for microstructural analyses. A solution of 70 vol.% phosphoric acid and 30 vol.% water was used as the etchant. Electrolytical etching was performed under the voltage of 5 V for 6–8 s. Microstructural characterization was performed using a Keyence VHX 6000 optical microscope (Osaka, Japan), and a ZEISS Merlin scanning electron microscope (Hallbergmoos, Germany), which was also used for EBSD tests.

## 3. Results and Discussion

The as-printed specimen presents a macro-morphology of multiple consecutive bands, shown in Figure 1. A total of 53 bands is observed in the specimen, indicating that the average height of one band is approximately 1.13 mm, corresponding to one cycle of 56 20-µm-thick layers. This shows that the morphology is related to the process parameters. 

**Figure 2:**
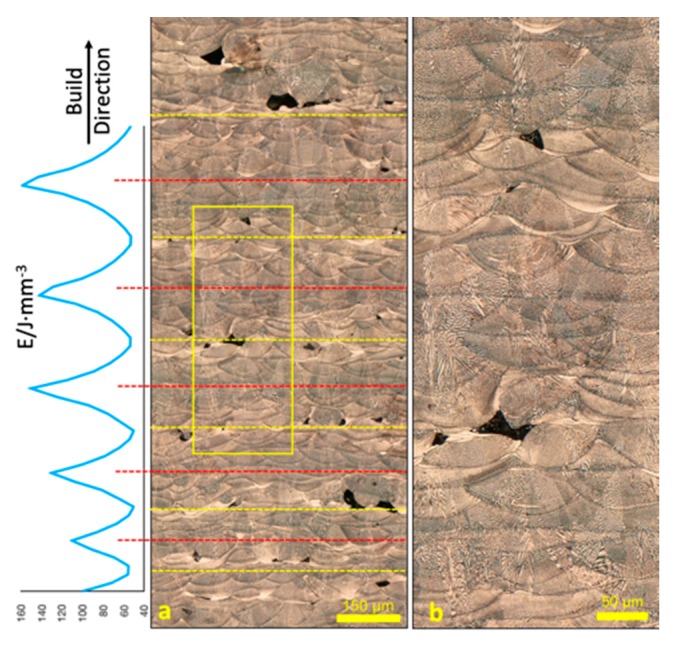
(**a**) Optical micrograph of the x-z plane corresponding to the variation of laser energy input and (**b**) detailed optical micrograph of the region in the yellow rectangle.

The optical micrograph of x-z plane of the specimen (along the build direction) corresponding to varying laser energy inputs is shown in Figure 2a, which clearly demonstrates that soundness of the specimen directly varied with the laser energy density. High laser energy density resulted in soundness, larger, regularly shaped melt pools, while low laser energy density led to porosity, un-melted powder, smaller, irregularly shaped melt pools. Good stacking and overlap of melt pools was found in regions with high energy input, while significant mismatch of melt pools, between adjacent layers tracks, was found under low energy input. The magnified image of the yellow rectangle in Figure 2a is shown in Figure 2b. As can be seen, the widths of the melt pools are approximately 100–120 µm, and 20–40 µm in depth, well in accordance with the hatch spacing and layer thickness. It is also observed that the overall shape of the melt pools in regions with higher energy inputs are larger in both the width and the depth, which means that increased laser energy will melt more extensively into the metal powder bed both horizontally and vertically. 

During SLM, low energy input cannot fully melt the metal powders, leaving some un-melted or partially melted powders in the as-printed specimen. Meanwhile, low energy input also leads to poor flowability in the molten metal, making it unable to spread out evenly in the present layer. Additionally, this further causes a relatively rugged surface, hindering the swiping of next layer’s powder, and leads to possible bridging among powder particles. In layers under continuous low energy inputs, pores can extend to 2–3 layers. However, in layers above those large pores where laser energy input rises, the morphology tends to be smoother, because this increase leads to better flowability of the molten metal, which spreads out more evenly in the current layer, smoothing the surface and improving the spreading of powders in the next layer. Thus, after a few layers with increasing energy inputs, the morphology of the specimen shifts to soundness. Hence, it is possible that the micromorphology of the SLM processed specimens can be altered by increasing or decreasing the laser energy inputs in different layers. This lends insight into controlling the soundness level in selective laser melting by a set of intentionally arranged energy inputs for different layers. Meanwhile, different types of micromorphology were obtained in one specimen, demonstrating the capability of achieving combinations or functional gradients of porous and sound zones in one component using selective laser melting, which would otherwise be improbable with other traditional manufacturing methods. 

**Figure 3:**
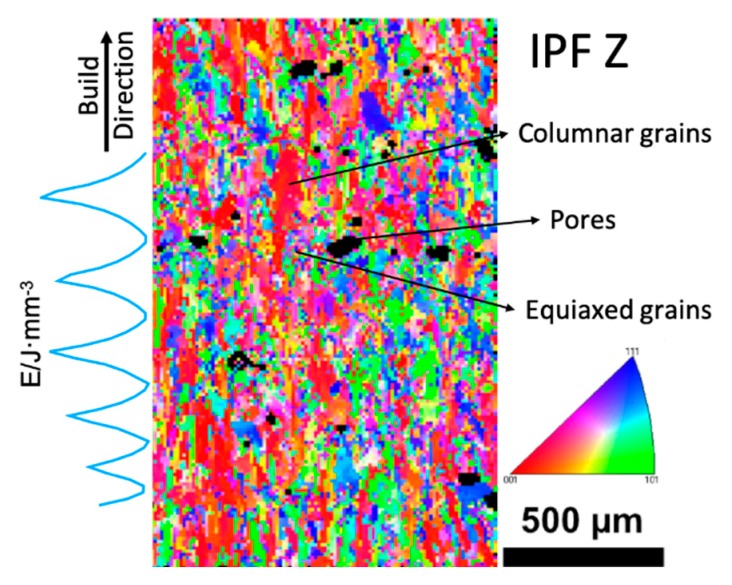
EBSD pattern of the x-z section.

Inverse pole figure (IPF) of the EBSD test of the x-z section is shown in Figure 3. In sound regions with high laser energy inputs, grains evolved into columnar grains along the build direction. Whereas, in porous regions indicating lower energy input, grains grew in an equiaxed pattern. 

Under high energy input, the temperature gradient was more significant, facilitating columnar grain growth. Also, the stacking of melt pools was in good order and the previous layer was re-melted more deeply under a higher laser energy input. This deeper re-melt caused grains in the previous layers to expose preferable crystal planes, acting as nucleation cites. Grains in the current layer tended to grow along the same orientation in those exposed partially re-melted grains, extending the grain through the melt pool boundary. Thus, grains in the center of melt pools can grow continuously through multiple layers along the build direction. As for regions with low energy input, both the temperature and temperature gradient were smaller. Metals in the previous layer were not re-melted sufficiently. Hence, nucleation cites mostly came from local melt pools, leading to the formation of equiaxed grains. This is in good agreement with other studies of SLM processed Inconel 718 alloys [16], where higher laser power facilitated columnar grain growth. Similar results have also been found in other SLM processed alloys [9]. 

**Figure 4:**
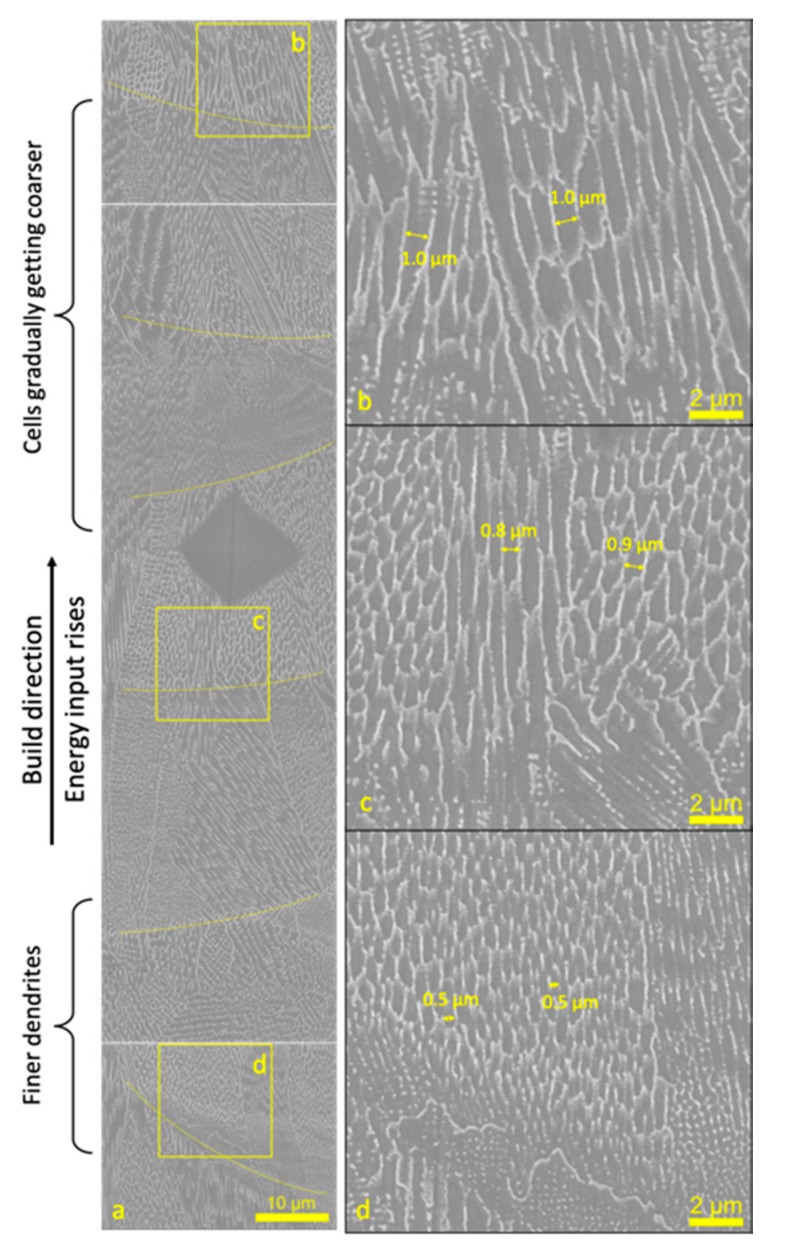
SEM images of the x-z section of the sample, (**a**) an assembly along the build direction showing several layers of melt pools; (**b**–**d**) magnified images.

An assembly of the SEM images of the x-z section along the build direction is shown in Figure 4a. A typical cellular dendritic growth is observed, which is also mentioned in references [7,17], as well as in other SLM processed alloys [3,18]. During a selective laser melting process, molten metal solidifies so rapidly with a cooling rate of up to 10^6^ K/s that there is no time for secondary dendrite arms to grow. Thus, only cellular growth appeared. Melt pool boundaries are depicted in the image. It can be seen that the cell sizes varied in different layers. A hardness measurement point was used as a locating spot to match the energy inputs in these layers. As the energy input rose, the size of cellular dendrites coarsened gradually. Shown in magnified images, the widths of the cellular dendrites in regions with high energy inputs are approximately 0.8–1.0 µm (Figure 4b,c), greater than that in regions with low energy inputs (~0.5 µm, Figure 4d). 

**Figure 5:**
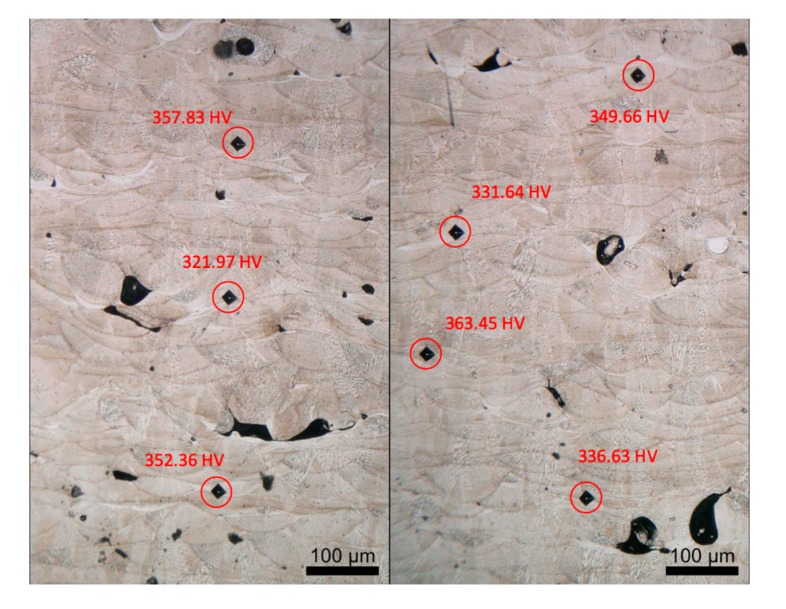
Microhardness test results.

Microhardness test results are shown in Figure 5. Overall the average microhardness value of the as-printed specimen is 344.79 ± 15.05 HV, comparable to the values reported in literature with samples fabricated using uniform parameters [6,19,20] (ranging from 325 to 395.8 HV for the as-printed specimens). However, it can be seen that the microhardness values varied slightly in different places. Generally, test points near pores displayed lower microhardness values than those tested in sound regions. This shows that higher energy inputs, corresponding to sound regions, lead to relatively higher hardness. This is because, when the laser beam scans the current layer, there is a heat affected zone in the previous layer where the metal is heated up below its melting point and cooled down. This process can be viewed as a solution heat treatment that enhances the microhardness of the affected zone. Under higher laser energy inputs, the previous layer goes through a more thorough heat treatment, hence, the microhardness tends to be higher. 

## 4. Conclusions

To explore the maneuverability of layer-by-layer variation of parameters during selective laser melting, a cycle of 56 layers with varying laser power and scanning speed was realized in an SLM processed Inconel 718 cuboid specimen with 53 cycles. Layer-wise variations of microstructure were acquired, showing the capability of microstructural control. The gradual shifts between soundness and porosity are observed. High energy density leads to sound regions with bigger melt pools and coarser cells, while low energy density results in smaller melt pools and cells, as well as more porosities. Additionally, high energy inputs boost columnar grain growth, while low energy inputs favor equiaxed grain growth. Different types of micromorphology concerning porous and sound regions, as well as different types of grain morphology were realized in a single specimen, which indicates that SLM is capable of achieving combinations or functional gradients of porous and sound zones or different distributions of columnar and equiaxed grains in one component. This provides a huge range of possibilities in the processing design of metal manufacturing to realize gradient structure for gradient function and high throughput microstructure-mechanical properties test. 

The as-printed specimens display a comparable microhardness than the ones fabricated using uniform parameters. With the increase of laser energy density, the specimen shifts from porous region to sound region within several layers. This provides insights for the attentive control of microstructures in selective laser melting. By increasing the laser energy input, layers with porosity can gradually transfer into layers with microstructural soundness.
